# Temporary Internal Fixation of Compound Fractures

**Published:** 1919-01

**Authors:** 


***Temporary Internal Fixation of Compound Fractures.*** By W. L. Brown and C. P. Brown. *Surgery, Gynecology and Obstetrics,* October, 1918.

In the treatment of gunshot fractures more and more stress is being laid upon the mechanical cleansing and immobilization of the wound.

As to immobilization, the authors consider that temporary internal fixation of compound fractures is liable to bring about most excellent results. This fixation should be realized preferably with the Parham-Martin band, or, if not possible, with a Lane's plate, and should be removed, under local anesthesia, at the end of 5 or 6 weeks, — as a rule as soon as sufficient callus and fibrous tissue have developed to hold the fragments in apposition.

The internal fixation foreign body has none of the objectionable features of the war missile, as to the possibility of developing infection. It is essentially aseptic, instead of being infected, and is always at a point where drainage is most efficient.

Immediate relief from pain, and great convenience of handling the limb for frequent dressings, are two of the main advantages of this technic.

The plates or bands do not interfere with bone regeneration or callus formation within the first 5 or 6 weeks, but they are liable to do this if they are left in longer. The plate or band is never covered by bone sufficiently to require a chisel or bone forceps for its removal at the end of 6 weeks.

Internal fixation proves particularly valuable in fractures of the humerus, certain fractures of the femur, and in cases in which both bones of the leg are fractured and comminuted.

If union fails after internal fixation, as it sometimes will, no such extensive deformity, overriding, and angulation, which often occur with external splinters, are to be feared.

The Parham band is much to be preferred to the Lane's plate, whenever it is appliable, since it does not need the perforating of the marrow cavity.

If plates are used, the screw holes should always be carefully curetted when the screws are removed.

In all cases, as complete external immobilization should be maintained as would be if internal fixation were not used.

